# A multivariate Poisson-log normal mixture model for clustering transcriptome sequencing data

**DOI:** 10.1186/s12859-019-2916-0

**Published:** 2019-07-16

**Authors:** Anjali Silva, Steven J. Rothstein, Paul D. McNicholas, Sanjeena Subedi

**Affiliations:** 1 Department of Mathematics and Statistics, University of Guelph, Guelph, N1G 2W1 Canada; 2 Department of Molecular and Cellular Biology, University of Guelph, Guelph, N1G 2W1 Ontario Canada; 3 Department of Mathematics and Statistics, McMaster University, Hamilton, L8S 4K1 Ontario Canada; 4 Department of Mathematical Sciences, Binghamton University, Binghamton, 13902 New York USA

**Keywords:** Clustering, RNA sequencing, Discrete data, Multivariate Poisson-log normal distribution, Markov chain Monte Carlo, Co-expression networks

## Abstract

**Background:**

High-dimensional data of discrete and skewed nature is commonly encountered in high-throughput sequencing studies. Analyzing the network itself or the interplay between genes in this type of data continues to present many challenges. As data visualization techniques become cumbersome for higher dimensions and unconvincing when there is no clear separation between homogeneous subgroups within the data, cluster analysis provides an intuitive alternative. The aim of applying mixture model-based clustering in this context is to discover groups of co-expressed genes, which can shed light on biological functions and pathways of gene products.

**Results:**

A mixture of multivariate Poisson-log normal (MPLN) model is developed for clustering of high-throughput transcriptome sequencing data. Parameter estimation is carried out using a Markov chain Monte Carlo expectation-maximization (MCMC-EM) algorithm, and information criteria are used for model selection.

**Conclusions:**

The mixture of MPLN model is able to fit a wide range of correlation and overdispersion situations, and is suited for modeling multivariate count data from RNA sequencing studies. All scripts used for implementing the method can be found at https://github.com/anjalisilva/MPLNClust.

**Electronic supplementary material:**

The online version of this article (10.1186/s12859-019-2916-0) contains supplementary material, which is available to authorized users.

## Background

RNA sequencing (RNA-seq) is used to determine the transcriptional dynamics of a biological system by measuring the expression levels of thousands of genes simultaneously [1, 2]. This technique provides counts of reads that can be mapped back to a biological entity, such as a gene or an exon, which is a measure of the gene’s expression under experimental conditions. Analyzing RNA-seq data is challenged by several factors, including the nature of the data, which is characterized by high dimensionality, skewness, and presence of a dynamic range that may vary from zero to over a million counts. Further, multivariate count data from RNA-seq is generally overdispersed. Upon obtaining raw counts of reads from an RNA-seq study, a typical bioinformatics analysis pipeline involves trimming, mapping, summarizing, normalizing and downstream analysis [3]. Cluster analysis is often performed as part of downstream analysis to identify key features between observations.

Clustering algorithms can be classified into two broad categories: distance-based or model-based approaches [4]. Distance-based clustering techniques include hierarchical clustering and partitional clustering [4]. Distance-based approaches utilize a distance function between pairs of data objects and group similar objects together into clusters. Model-based approaches involve clustering data objects using a mixture-modeling framework [4–8]. Compared to distance-based approaches, model-based approaches offer better interpretability because the resulting model for each cluster directly characterizes that cluster [4]. In model-based approaches, the conditional probability of each data object belonging to a cluster is calculated.

The probability distribution function of a mixture model is $f(\boldsymbol {y}| \pi _{1}, \ldots, \pi _{G},\boldsymbol {\vartheta }_{1}, \ldots, \boldsymbol {\vartheta }_{G}) =\!\! \sum \nolimits _{g=1}^{G} \pi _{g} f_{g}(\boldsymbol {y} | \boldsymbol {\vartheta }_{g})$, where *G* is the total number of clusters, *f*_*g*_(·) is the distribution function with parameters ***𝜗***_*g*_, and *π*_*g*_>0 is the mixing weight of the *g*^th^ component such that $\sum \nolimits _{g=1}^{G} \pi _{g}=1$. An indicator variable *z*_*ig*_ is used for cluster membership, such that *z*_*ig*_ equals 1 if the *i*th observation belongs to component *g* and 0 otherwise. The predicted cluster memberships at the maximum likelihood estimates of the model parameters are given by the maximum *a posteriori* probability, MAP$(\hat {z}_{ig}$). The $\text {MAP}(\hat {z}_{ig}) = 1$ if $\arg \max _{h}\{\hat {z}_{ih}\}=g$ and $\text {MAP}(\hat {z}_{ig}) = 0$ otherwise. Parameter estimation is typically carried out using maximum likelihood algorithms, such as the expectation-maximization (EM) algorithm [9]. The parameter estimation methods are fitted for a range of possible number of components and the optimal number is selected using a model selection criterion. Typically, one component represents one cluster [8].

Clustering of gene expression data allows identifying groups of genes with similar expression patterns, called gene co-expression networks. Inference of gene networks from expression data can lead to better understanding of biological pathways that are active under experimental conditions. This information can also be used to infer the biological function of genes with unknown or hypothetical functions based on their cluster membership with genes of known functions and pathways [10]. Over the past few years, a number of mixture model-based clustering approaches for gene expression data from RNA-seq studies have emerged based on the univariate Poisson and negative binomial (NB) distributions [11–13]. Although these distributions seem a natural fit to count data, there can be limitations when applied in the context of RNA-seq as outlined in the following paragraph.

The Poisson distribution is used to model discrete data, including expression data from RNA-seq studies. However, the multivariate extension of the Poisson distribution can be computationally expensive. As a result, the univariate Poisson distribution is often utilized in clustering algorithms, which leads to the assumption that samples are independent conditionally on the components [11, 12, 14]. This assumption is unlikely to hold in real situations. Further, the mean and variance coincide in the Poisson distribution. As a result, the Poisson distribution may provide a good fit to RNA-seq studies with a single biological replicate across technical replicates [15]. However, current RNA-seq studies often utilize more than one biological replicate in order to estimate the biological variation between treatment groups. In such studies, RNA-seq data exhibit more variability than expected (called “overdispersion”) and the Poisson distribution may not provide a good fit for the data [15, 16]. Due to the smaller variation predicted by Poisson distribution, type-I errors in the data can be underestimated [16]. The use of NB distribution may alleviate some of these issues as the mean and variance differ. However, NB can fail to provide a good fit to heavy tailed data like RNA-seq [17].

The multivariate Poisson-log normal (MPLN) distribution [18] is a multivariate log normal mixture of independent Poisson distributions. It is a two-layer hierarchical model, where the observed layer is a multivariate Poisson distribution and the hidden layer is a multivariate Gaussian distribution [18, 19]. The MPLN distribution is suitable for analyzing multivariate count measurements and offers many advantages over other discrete distributions [20, 21]. Importantly, the hidden layer of the MPLN distribution is a multivariate Gaussian distribution, which allows for the specification of a covariance structure. As a result, independence no longer needs to be assumed between variables. The MPLN distribution can also account for overdispersion in count data and supports negative and positive correlations, unlike other multivariate discrete distributions such as multinomial or negative multinomial [22].

Here, a novel mixture model-based clustering method is presented for RNA-seq using MPLN distributions. The proposed clustering technique is explored in the context of clustering genes. The performance of the method is evaluated through data-driven simulations and real data.

## Results

### Transcriptome data analysis

To illustrate the applicability of mixtures of MPLN distributions, it is applied to a RNA-seq dataset. For comparison purposes, three model-based clustering methods were also used. These include HTSCluster [11, 14], Poisson.glm.mix [12] and MBCluster.Seq [13]. Poisson.glm.mix offers three different parameterizations for the Poisson mean, which will be termed m = 1, m = 2, and m = 3. MBCluster.Seq offers clustering via mixtures of Poisson, termed MBCluster.Seq, Poisson, and clustering via mixtures of NB, termed MBCluster.Seq, NB.

Typically, only a subset of differentially expressed genes is used for cluster analysis. Normalization factors representing library size estimate for samples for all methods were obtained using trimmed mean of *M* values (TMM) [23, 24] from the calcNormFactors function of edgeR package. Initialization is done via *k*-means for HTSCluster and MBCluster.Seq. An option to specify normalization or initialization method was not available for Poisson.glm.mix, thus default settings were used. Note, for MBCluster.Seq, *G*=1 cannot be run.

In the context of real data clustering, it is not possible to compare the clustering results obtained from each method to a ‘true’ clustering of the data as such classification does not exist. To identify if co-expressed genes are implicated in similar biological processes, functions or components, an enrichment analysis was performed on the gene clusters using the Singular Enrichment Analysis tool available on AgriGO [25]. Singular Enrichment Analysis tool identifies enriched gene ontology (GO) terms provided a list of gene identifiers by comparing it to a background population or reference from which the query list is derived [25]. A significance level of 5% is used with Fisher statistical testing and Yekutieli multi-test adjustment. GO defines three distinct ontologies, called biological process, molecular function, and cellular component.

### Transcriptome data analysis: cranberry bean RNA-seq data

In the study by Freixas-Coutin et al. [26], RNA-seq was used to monitor transcriptional dynamics in the seed coats of darkening (D) and non-darkening (ND) cranberry beans (*Phaseolus vulgaris* L.) at three developmental stages: early (E), intermediate (I) and mature (M). A summary of this dataset is provided in Table 1. The aim of their study was to evaluate if the changes in the seed coat transcriptome were associated with proanthocyanidin levels as a function of seed development in cranberry beans. For each developmental stage, 3 biological replicates were considered for a total of 18 samples. The RNA-seq data are available on the National Center for Biotechnology Information (NCBI) Sequence Read Archive (SRA) under the BioProject PRJNA380220. The study identified 1336 differentially expressed genes, which were used for the cluster analysis.

**Table 1:** Summary of the cranberry bean RNA-seq dataset used for cluster analysis

No. of genes	Replicates per condition	Read count range	5-95% Read count range	Library size range	Platform & Instrument
1336	(3,3,3,3,3,3)	(0–483,965)	(205–3652)	(937,559–1,870,947)	Illumina HiSeq 2500

The raw read counts for genes were obtained from Binary Alignment/Map files using samtools [27] and HTSeq [28]. The median value from the 3 replicates per each developmental stage was chosen. In the first run, *T*_1_, data was clustered for a range of *G*=1,…,11 using *k*-means initialization with 3 runs. (Note, for MBCluster.Seq, *G*=1 cannot be run.) Since model selection criteria selected *G*=2 or *G*=11 for HTSCluster, Poisson.glm.mix, and MBCluster.Seq, further clustering runs were performed for these methods using ranges of *T*_2_ :*G*=1,…,20; *T*_3_ :*G*=1,…,30; *T*_4_ :*G*=1,…,40; *T*_5_ :*G*=1,…,50 and *T*_6_ :*G*=1,…,100. The clustering results are summarized in Table 2. Note, more than 10 models need to be considered for applying slope heuristics, dimension jump (Djump) and data-driven slope estimation (DDSE), and because *G*=1 cannot be run for MBCluster.Seq, slope heuristics could not be applied for *T*_1_.

**Table 2:** Number of clusters selected using different model selection criteria for the cranberry bean RNA-seq dataset for *T*_1_ to *T*_6_

Method	BIC	ICL	AIC	AIC3	Djump	DDSE	BIC	ICL	AIC	AIC3	Djump	DDSE
	*T*_1_:*G*=1,…,11						*T*_2_:*G*=1,…,20					
mixtures of MPLN	4	4	5	4	2	2	-	-	-	-	-	-
HTSCluster	11	11	11	11	8	8	20	20	20	20	11	11
Poisson.glm.mix, m = 1	11	11	11	11	2	7	20	20	20	20	9	9
Poisson.glm.mix, m = 2	11	11	11	11	8	8	20	20	20	20	8	8
Poisson.glm.mix, m = 3	11	11	11	11	8	8	20	20	20	20	12	10
MBCluster.Seq, Poisson	11	11	11	11	-	-	14	14	20	16	8	15
MBCluster.Seq, NB	2	2	2	2	-	-	2	2	2	2	7	14
	*T*_3_:*G*=1,…,30						*T*_4_:*G*=1,…,40					
HTSCluster	30	30	30	30	16	16	40	40	40	40	22	22
Poisson.glm.mix, m = 1	30	30	30	30	10	10	40	40	40	40	29	29
Poisson.glm.mix, m = 2	30	30	30	30	19	20	40	40	40	40	18	18
Poisson.glm.mix, m = 3	30	30	30	30	13	13	40	40	40	40	24	24
MBCluster.Seq, NB	2	2	2	2	7	19	2	2	2	2	22	22
	*T*_5_:*G*=1,…,50						*T*_6_:*G*=1,…,100					
HTSCluster	50	50	50	50	22	22	100	100	100	100	41	76
Poisson.glm.mix, m = 1	50	50	50	50	30	30	100	100	100	100	24	34
Poisson.glm.mix, m = 2	50	50	50	50	29	29	100	100	100	100	40	40
Poisson.glm.mix, m = 3	50	50	50	50	17	17	100	100	100	100	45	45
MBCluster.Seq, NB	2	2	2	2	22	30	2	2	2	2	42	47

**Fig. 1:**
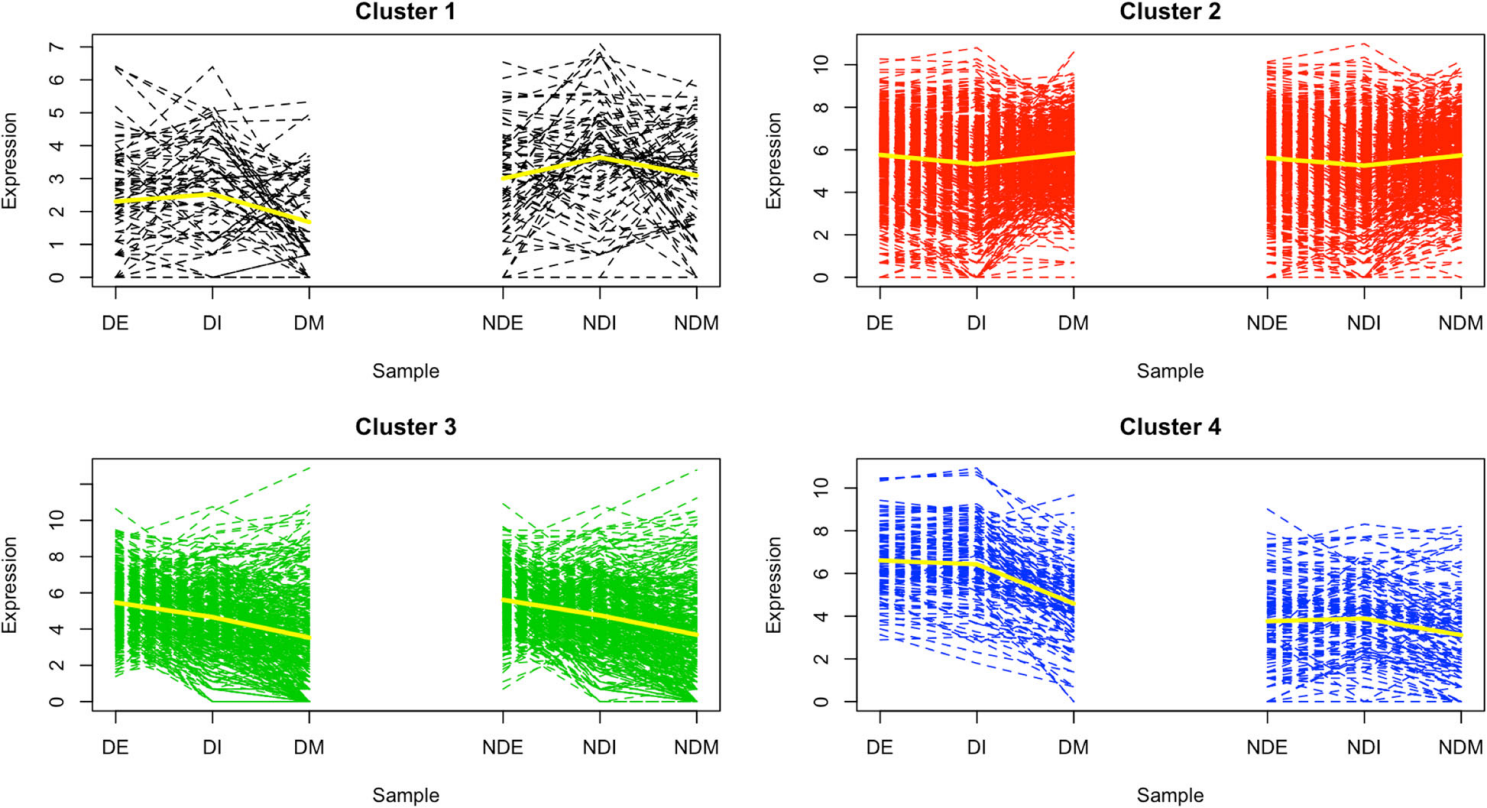
The expression patterns for the *G*=4 model for the cranberry bean RNA-seq dataset clustered using mixtures of MPLN distributions. The expression represents the log-transformed counts. The yellow line represents the mean expression level for each cluster

For the mixtures of MPLN distributions, all information criteria selected a model with *G*=4, with the exception of the AIC, which selected a *G*=5 model in *T*_1_. Recall that the AIC is known to favor more complex models with more parameters. A cross tabulation comparison of *G*=4 model with that of *G*=5 did not reveal any significant patterns, but rather random classification results were observed. For the *G*=4 model, each cluster contained 71, 731, 415 and 119 genes respectively, and the expression patterns of these models are provided in Fig. 1. For MBCluster.Seq, NB, a model with *G*=2 was selected. This is the lowest cluster size considered in the range of clusters for this method as *G*=1 cannot be run for MBCluster.Seq. For *G*=2 model, Cluster 1 contained 467 genes and Cluster 2 contained 869 genes (expression patterns provided in Additional file 1: Figure S1). A comparison of this model with that of *G*=4, from mixtures of MPLN distributions, did not reveal any significant patterns. For all other methods in *T*_1_, information criteria selected *G*=11.

During *T*_2_, a model with *G*=14 was selected for MBCluster.Seq, Poisson by the BIC and ICL (expression patterns provided in Additional file 1: Figure S2). A comparison of this model with that of *G*=4, from mixtures of MPLN distributions, did not reveal any significant patterns. With further runs (*T*_3_,…,*T*_6_), it was evident that the highest cluster size is selected for HTSCluster and Poisson.glm.mix. No changes were observed for MBCluster.Seq, NB, as the lowest cluster size, *G*=2, is selected. All information criteria (BIC, ICL, AIC, AIC3) gave similar results, suggesting a high degree of certainty in the assignment of genes into clusters, i.e., that the posterior probabilities $\hat {z}_{ig}$ are generally close to zero or one. The results from slope heuristics (Djump and DDSE) highly varied across *T*_1_,…,*T*_6_. For this reason, overfitting and underfitting methods were run for *G*=1,…,100, as in *T*_6_, but for 20 different times. Results for both information criteria and slope heuristics are provided in Table 3. The results from slope heuristics highly varied across the 20 different clustering runs, as evident by the large range in the number of models selected.

**Table 3:** Range of clusters selected using different model selection criteria for the cranberry bean RNA-seq dataset for *T*_6_, repeated 20 times

	BIC		ICL		AIC		AIC3	
Method	Range	Breakdown	Range	Breakdown	Range	Breakdown	Range	Breakdown
HTSCluster	97–100	97(1); 99(4); 100 (15)	97–100	97(1); 99(4); 100 (15)	100–100	100(20)	99–100	99(2); 100(18)
Poisson.glm.mix, m = 1	100–100	100(20)	100–100	100(20)	100–100	100(20)	100–100	100(20)
Poisson.glm.mix, m = 2	99–100	99(1); 100(19)	99–100	99(1); 100(19)	99–100	99(1); 100(19)	99–100	99(1); 100(19)
Poisson.glm.mix, m = 3	100–100	100(20)	100–100	100(20)	100–100	100(20)	100–100	100(20)
MBCluster.Seq, NB	2–2	2(20)	2–2	2(20)	2–2	2(20)	2–2	2(20)
	Djump							
Method	Range	Breakdown						
HTSCluster	36–76	36(1); 38(1); 43(1); 44(3); 46(1); 47(1); 49(2); 50(2); 51(3); 54(2); 63(1); 68(1); 76(1)						
Poisson.glm.mix, m = 1	21–74	21(1); 24(1); 29(1); 35(1); 37(1); 38(1); 40(1); 42(1); 44(1); 45(1); 47(1); 49(1); 56(1); 60(1); 63(2); 64(1); 66(1); 68(1); 74(1)						
Poisson.glm.mix, m = 2	20–68	20(1); 28(3); 33(1); 35(1); 38(1); 40(1); 44(1); 47(2); 49(1); 50(1); 53(1); 55(2); 60(2); 63(1); 68(1)						
Poisson.glm.mix, m = 3	23–77	23(1); 33(1); 35(2); 39(1); 40(1); 41(1); 42(1); 45(2); 47(1); 50(2); 52(1); 55(1); 56(1); 65(1); 67(1); 69(1); 77(1)						
MBCluster.Seq, NB	28–66	28(2); 29(1); 38(1); 39(1); 42(4); 46(1); 47(1); 51(1); 52(1); 55(1); 57(1); 58(1); 59(1); 64(1); 65(1); 66(1)						
	DDSE							
Method	Range	Breakdown						
HTSCluster	22–63	22(1); 29(2); 36(1); 37(1); 38(1); 41(1); 43(1); 44(3); 46(1); 47(1); 49(2); 50(1); 51(2); 54(1); 63(1)						
Poisson.glm.mix, m = 1	33–77	33(1); 34(1); 43(1); 46(1); 47(1); 49(1); 50(1); 52(1); 54(1); 56(1); 59(2); 60(1); 63(2); 65(1); 66(1); 67(1); 70(1); 77(1);						
Poisson.glm.mix, m = 2	33–87	33(1); 40(1); 47(1); 49(1); 53(1); 54(1); 55(1); 59(1); 60(3); 63(1); 66(1); 68(1); 70(1); 71(1); 74(2); 83(1); 87(1)						
Poisson.glm.mix, m = 3	36–71	36(1); 40(1); 42(2); 44(1); 45(1); 46(2); 47(1); 48(1); 49(1); 50(2); 52(1); 56(1); 61(1); 64(1); 65(1); 69(1); 71(1)						
MBCluster.Seq, NB	44–70	44(1); 46(2); 47(3); 51(1); 53(1); 54(1); 55(2); 56(1); 57(3); 58(1); 59(1); 62(2); 70(1)						

Due to model selection issues with over and under fitting, downstream analysis was only conducted using the *G*=4 model of mixtures of MPLN distributions, *G*=14 model of MBCluster.Seq, Poisson, and *G*=2 model of MBCluster.Seq, NB. The GO enrichment analysis results for all models are provided in Additional file 2. Only $\frac {1}{2}, \frac {3}{4}$, and $\frac {5}{14}$ clusters contained enriched GO terms in *G*=2,*G*=4, and *G*=14 models, respectively. Among the models, clear expression patterns were evident for the *G*=14 model, and this can be attributed to the fact that there are more clusters present in this model. However, only 5 of the 14 clusters exhibited significant GO terms.

Further analysis was only conducted on the *G*=4 model of the mixtures of MPLN distributions, because comparing the cluster composition of genes across different methods, with respect to biological context, is beyond the scope of this article. For the *G*=4 model, Cluster 1 genes were highly expressed in intermediate developmental stage, compared to other developmental stages, regardless of the variety (see Figure 1). The GO enrichment analysis identified genes belonging to pathogenesis, multi-organism process and nutrient reservoir activity (see Additional file 2). For Cluster 2, no GO terms exhibited enrichment and the expression of genes might be better represented by two or more distinct clusters.

Cluster 3 genes showed higher expression in early developmental stage, compared to other developmental stages, regardless of the variety. Here, genes belonged to oxidoreductase activity, enzyme activity, binding and dehydrogenase activity. Finally, Cluster 4 genes were more highly expressed in the darkening variety relative to the non-darkening variety. The GO enrichment analysis identified Cluster 4 genes as containing biosynthetic genes. Further examination identified that many of these genes were annotated as flavonoid/proanthocyanidin biosynthesis genes in the *P. vulgaris* genome. Polyphenols, such as proanthocyanidins, are synthesized by the phenylpropanoid pathway and are found on seed coats (Reinprecht et al. 2013). Proanthocyanidins have been shown to convert from colorless to visible pigments during oxidation [29]. Beans with regular darkening of seed coat color is known to have higher levels of polyphenols compared to beans with slow darkening [29, 30].

### Simulation data analysis: mixtures of MPLN distributions

To simulate data that mimics real data, the library sizes and count ranges in simulated datasets were ensured to be within the same 5–95% ranges as those observed for real data. For the simulation study, three different settings were considered. In simulations 1 and 2, 50 datasets with one underlying cluster and 50 datasets with two underlying clusters were generated, respectively. In simulation 3, 30 datasets with three underlying clusters were generated. All datasets had *n*=1000 observations and *d*=6 samples generated using mixtures of MPLN distributions. The covariance matrices for each setting were generated using the genPositiveDefMat function in clusterGeneration package, with a range specified for variances of the covariance matrix [31].

Comparative studies were conducted to evaluate the ability to recover the true underlying number of clusters. For this purpose, the following model-based methods were used: HTSCluster, Poisson.glm.mix and MBCluster.Seq. Initialization of *z*_*ig*_ for all methods was done using the *k*-means algorithm with 3 runs. For simulation 1, *π*_1_=1 and a clustering range of *G*=1,…,3 was considered. For simulation 2, *π*_1_=0.79 and a clustering range of *G*=1,…,3 was considered. For simulation 3, *π*_1_=0.3 and *π*_2_=0.5, and a clustering range of *G*=2,…,4 was considered. In addition to model-based methods, three distance-based methods were also used: *k*-means [32], partitioning around medoids [33] and hierarchical clustering. These were only applied to simulation 2 and simulation 3. Further, a graph-based method employing Louvain algorithm [34] was also used. The parameter estimation results for the mixtures of MPLN algorithm are provided in Additional file 3. The clustering results for all methods are summarized in Table 4.

**Table 4:** Number of clusters selected (average ARI, standard deviation) for each simulation setting using mixtures of MPLN distributions

Setting	Method	BIC	ICL	AIC	AIC3	None
1	mixtures of MPLN	1 (1.00, 0.00)	1 (1.00, 0.00)	1 (1.00, 0.00)	1 (1.00, 0.00)	-
	HTSCluster	3 (0.00, 0.00)	3 (0.00, 0.00)	3 (0.00, 0.00)	3 (0.00, 0.00)	-
	Poisson.glm.mix, m = 1	3 (0.00, 0.00)	3 (0.00, 0.00)	3 (0.00, 0.00)	3 (0.00, 0.00)	-
	Poisson.glm.mix, m = 2	3 (0.00, 0.00)	3 (0.00, 0.00)	3 (0.00, 0.00)	3 (0.00, 0.00)	-
	Poisson.glm.mix, m = 3	1-3 (1.00, 0.00)	1-3 (1.00, 0.00)	1-3 (1.00, 0.00)	1-3 (1.00, 0.00)	-
	MBCluster.Seq, Poisson	-	-	-	-	-
	MBCluster.Seq, NB	-	-	-	-	-
	Louvain	-	-	-	-	3-5 (0.00, 0.00)
2	mixtures of MPLN	2 (1.00, 0.00)	2 (1.00, 0.00)	2 (1.00, 0.00)	2 (1.00, 0.00)	-
	HTSCluster	3 (-0.01, 0.01)	3 (-0.01, 0.01)	3 (-0.01, 0.01)	3 (-0.01, 0.01)	-
	Poisson.glm.mix, m = 1	3 (0.09, 0.04)	3 (0.09, 0.04)	3 (0.09, 0.04)	3 (0.09, 0.04)	-
	Poisson.glm.mix, m = 2	3 (0.00, 0.02)	3 (0.00, 0.02)	3 (0.00, 0.02)	3 (0.00, 0.02)	-
	Poisson.glm.mix, m = 3	1-3 (0.00, 0.01)	1-3 (0.00, 0.01)	1-3 (0.00, 0.01)	1-3 (0.00, 0.01)	-
	MBCluster.Seq, Poisson	3 (0.00, 0.01)	3 (0.00, 0.01)	3 (0.00, 0.01)	3 (0.00, 0.01)	-
	MBCluster.Seq, NB	2 (-0.01, 0.06)	2 (-0.01, 0.06)	2 (-0.01, 0.06)	2 (-0.01, 0.06)	-
	Kmeans	-	-	-	-	2 (-0.06, 0.03)
	Medoids	-	-	-	-	2 (0.70, 0.03)
	Hierarchical	-	-	-	-	2 (-0.00, 0.008)
	Louvain	-	-	-	-	3-8 (0.014, 0.01)
3	mixtures of MPLN	3 (0.99, 0.01)	3 (0.99, 0.01)	3 (0.99, 0.01)	3 (0.99, 0.01)	-
	HTSCluster	4 (0.02, 0.02)	4 (0.02, 0.02)	4 (0.02, 0.02)	4 (0.02, 0.02)	-
	Poisson.glm.mix, m = 1	4 (0.15, 0.03)	4 (0.15, 0.03)	4 (0.15, 0.03)	4 (0.15, 0.03)	-
	Poisson.glm.mix, m = 2	4 (0.04, 0.02)	4 (0.04, 0.02)	4 (0.04, 0.02)	4 (0.04, 0.02)	-
	Poisson.glm.mix, m = 3	2-4 (0.02, 0.01)	2-4 (0.02, 0.01)	2-4 (0.02, 0.01)	2-4 (0.02, 0.01)	-
	MBCluster.Seq, Poisson	4 (0.02, 0.01)	4 (0.02, 0.01)	4 (0.02, 0.01)	4 (0.02, 0.01)	-
	MBCluster.Seq, NB	2 (0.00, 0.01)	2 (0.00, 0.01)	2 (0.00, 0.01)	2 (0.00, 0.01)	-
	Kmeans	-	-	-	-	3 (0.03, 0.11)
	Medoids	-	-	-	-	3 (0.42, 0.07)
	Hierarchical	-	-	-	-	3 (-0.00, 0.07)
	Louvain	-	-	-	-	5-7 (0.015, 0.01)

The adjusted Rand index (ARI) values obtained for mixtures of MPLN were equal to or very close to one, indicating that the algorithm is able to assign observations to the proper clusters, i.e., the clusters that were originally used to generate the simulation datasets. Note, for MBCluster.Seq, *G*=1 cannot be run, and the corresponding row of results has been left blank on Table 4. Although a range of clusters *G*=1,2,3 was selected for Poisson.glm.mix, m = 3 in simulation 1, an ARI value of one was obtained because all runs resulted in only one cluster (others were empty clusters). Distance-based methods and the graph-based method resulted in low ARI values.

### Simulation data analysis: mixtures of negative binomial distributions

In this simulation, 50 datasets with two underlying clusters were generated. All datasets had *n*=200 observations and *d*=6 samples generated using mixtures of negative binomial distributions. Comparative studies were conducted as specified earlier. Initialization of *z*_*ig*_ for all methods was done using the *k*-means algorithm with 3 runs. Here, *π*_1_=0.79 and a clustering range of *G*=1,…,3 was considered. The clustering results are summarized in Table 5. The ARI values obtained for mixtures of MPLN were equal to or very close to one, indicating that the algorithm is able to assign observations to the proper clusters. Low ARI values were observed for all other model-based clustering methods and the graph-based method. Interestingly, application of distance-based methods resulted in high ARI values.

**Table 5:** Number of clusters selected (average ARI, standard deviation) for the simulation setting using mixtures of negative binomial distributions

Method	BIC	ICL	AIC	AIC3	None
mixtures of MPLN	2 (1.00, 0.00)	2 (1.00, 0.00)	2-3 (0.99, 0.02)	2-3 (0.99, 0.02)	-
HTSCluster	2-3 (0.008, 0.02)	1 (0.00, 0.00)	3 (0.008, 0.02)	3 (0.008, 0.02)	-
Poisson.glm.mix, m = 1	1-3 (0.002, 0.02)	1 (0.00, 0.00)	3 (0.001, 0.01)	3 (0.001, 0.01)	-
Poisson.glm.mix, m = 2	2-3 (0.005, 0.02)	1 (0.00, 0.00)	2-3 (0.006, 0.02)	3 (0.006, 0.02)	-
Poisson.glm.mix, m = 3	1-3 (0.007, 0.02)	1 (0.00, 0.00)	3 (0.004, 0.02)	3 (0.004, 0.02)	-
MBCluster.Seq, Poisson	2 (0.005, 0.02)	2 (0.005, 0.02)	2 (0.005, 0.02)	2 (0.005, 0.02)	-
MBCluster.Seq, NB	2 (0.005, 0.01)	2 (0.005, 0.01)	2 (0.005, 0.01)	2 (0.005, 0.01)	-
Kmeans	-	-	-	-	2 (1.00, 0.00)
Medoids	-	-	-	-	2 (1.00, 0.00)
Hierarchical	-	-	-	-	2 (1.00, 0.00)
Louvain	-	-	-	-	7-9 (-0.0006, 0.005)

## Discussion

A model-based clustering technique for RNA-seq data has been introduced. The approach utilizes a mixture of MPLN distributions, which has not previously been used for model-based clustering of RNA-seq data. The transcriptome data analysis showed the applicability of mixture model-based clustering methods on RNA-seq data. Information criteria selected the highest cluster size considered in the range of clusters for HTSCluster and Poisson.glm.mix. For MBCluster.Seq, NB, the lowest cluster size considered in the range of clusters was selected. This could potentially imply that these mixtures of Poisson and NB models are not providing a good fit to the data. However, further research is needed in this direction, including the search for other model selection criteria. The GO enrichment analysis (p-value <0.05) identified enriched terms in 75% of the clusters resulting from mixtures of MPLN distributions, whereas only 50% of clusters from MBCluster.Seq, NB and 36% of the clusters from MBCluster.Seq, Poisson contained enriched GO terms.

Using simulated data from mixtures of MPLN distributions, it was illustrated that the algorithm for mixtures of MPLN distributions is effective and returned favorable clustering results. It was observed that other model-based methods from the current literature failed to identify the true number of underlying clusters a majority of the time. Clustering trends similar to those observed for transcriptome data analysis were observed for other model-based methods during the simulation data analysis. Distance-based methods failed to assign observations to proper clusters, as evident by the low ARI values. The graph-based method, Louvain, also failed to identify the true number of underlying clusters.

Using simulated data from mixtures of negative binomial distributions, it was illustrated that the algorithm for mixtures of MPLN distributions is effective and returned favorable clustering results. The distance-based methods also assigned observations to proper clusters resulting high ARI values. It was observed that other model-based methods from the current literature, as well as the graph-based method, failed to identify the true number of underlying clusters a majority of the time. Although the correct numbers of clusters were selected by MBCluster.Seq, proper cluster assignment has not taken place as evident by the low ARI values. Note that although MBCluster.Seq, NB is based on negative binomial distributions, it has low ARI (approx. 0). This could be because the implementation of the approach by [35] available in R package MBCluster.Seq at the moment only performs clustering based on the expression profiles. Si et al. [35] mention that clustering could be done according to both the overall expression levels and the expression profiles by some modification to the parameters, but the implementation of the approach was not available in the R package. Additionally, across all studies (both real and simulated) it is evident that *G*=2 is selected via information criteria, when MBCluster.Seq, NB is used for clustering.

Overall, the transcriptome data analysis together with simulation studies show superior performance of mixtures of MPLN distributions, compared to other methods presented.

## Conclusions

The mixture model-based clustering method based on MPLN distributions is an excellent tool for analysis of RNA-seq data. The MPLN distribution is able to describe a wide range of correlation and overdispersion situations, and is ideal for modeling RNA-seq data, which is generally overdispersed. Importantly, the hidden layer of the MPLN distribution is a multivariate Gaussian distribution, which accounts for the covariance structure of the data. As a result, independence does not need to be assumed between variables in clustering applications.

The scripts used to implement this approach are publicly available and reusable such that they can be simply modified and utilized in any RNA-seq data analysis pipeline. Further, the vector of library size estimates for samples can be relaxed and the proposed clustering approach can be applied to any discrete dataset. A direction for future work would be to investigate subspace clustering methods to overcome the *curse of dimensionality* as high-dimensional RNA-seq datasets become frequently available.

## Methods

### Mixtures of MPLN Distributions

The sequencing depth can differ between samples in an RNA-seq study. Therefore, the assumption of equal means across conditions is unlikely to hold. To account for the differences in library sizes across each sample *j*, a fixed, known constant, *s*_*j*_, representing the normalized library sizes is added to the mean of the Poisson distribution. Thus, for genes *i*∈{1,…,*n*} and samples *j*∈{1,…,*d*}, the MPLN distribution is modified to give

$$\begin{aligned} Y_{ij} | \theta_{ij} &\sim \mathscr{P}(\exp\{\theta_{ij}+\log s_{j}\})\\ (\theta_{i1}, \ldots,\theta_{id})^{\prime} &\sim \mathscr{N}_{d}(\boldsymbol{\mu},\boldsymbol{\Sigma}). \end{aligned} $$

A *G*-component mixture of MPLN distributions can be written

$${\begin{aligned} f(\mathbf{y};\boldsymbol{\Theta})&=\sum\limits_{g=1}^{G}\pi_{g}f_{\mathbf{Y}}(\mathbf{y}|\boldsymbol{\mu}_{g},\boldsymbol{\Sigma}_{g})\\ &= \sum\limits_{g=1}^{G}\pi_{g}\int_{\mathbb{R}^{d}} \left(\! \prod_{j=1}^{d} f(y_{ij}| \theta_{ijg}, s_{j})\!\right) ~f\!(\boldsymbol{\theta}_{ig}|\boldsymbol{\mu}_{g},\boldsymbol{\Sigma}_{g}\!)~d\boldsymbol{\theta}_{ig}, \end{aligned}} $$

where ***Θ***=(*π*_1_,…,*π*_*G*_,***μ***_1_,…,***μ***_*G*_,***Σ***_1_,…,***Σ***_*G*_) denotes all model parameters and *f*_**Y**_(**y**;***μ***_*g*_,***Σ***_*g*_) denotes the distribution of the *g*th component with parameters ***μ***_*g*_ and ***Σ***_*g*_. The unconditional moments of the MPLN distribution can be obtained via conditional expectation results and standard properties of the Poisson and log normal distributions. For a *G*-component mixture of MPLN distributions, the mean of *Y*_*j*_ is $\mathbb {E}(Y_{j}) = \exp \left \{\boldsymbol {\mu }_{jg} + \frac {1}{2} \sigma _{jjg} \right \} \overset {\text {def}}{=} \boldsymbol {m}_{jg}$ and the variance is $\mathbb {V}\text {ar}(Y_{j}) = \boldsymbol {m}_{jg} + \boldsymbol {m}_{jg}^{2} (\exp \{\sigma _{jjg}\} - 1)$. Here, *σ*_*jjg*_ represents the diagonal elements of ***Σ***_*g*_, for *j*=1,…,*d*. Now, $\mathbb {V}\text {ar}(Y_{j}) \geq \mathbb {E}(Y_{j})$ so there is overdispersion for the marginal distribution with respect to the Poisson distribution.

### Parameter Estimation

To estimate the parameters, a maximum likelihood estimation procedure based on the EM algorithm is used. In the context of clustering, the unknown cluster membership variable is denoted by **Z**_*i*_ such that *Z*_*ig*_=1 if an observation *i* belongs to group *g* and *Z*_*ig*_=0 otherwise, for *i*=1,…,*n*;*g*=1,…,*G*. The complete-data consist of (**y**,**z**,***θ***), the observed and missing data. Here, **z** is a realization of **Z**. The complete-data log-likelihood for the MPLN mixture model is

$$ {\begin{aligned} l_{c}(\boldsymbol{\Theta}) & \,=\, \sum\limits_{i=1}^{n} \sum\limits_{g=1}^{G} z_{ig} \log \pi_{g} \left(\sum\limits_{j=1}^{d} f(y_{ij}| \theta_{ijg}, s_{j})\right) ~f(\boldsymbol{\theta}_{ig}|\boldsymbol{\mu}_{g},\boldsymbol{\Sigma}_{g})\\ & = \sum\limits_{g=1}^{G} n_{g} \log \pi_{g} - \sum\limits_{i=1}^{n} \sum\limits_{g=1}^{G} \sum\limits_{j=1}^{d} z_{ig} \exp \{\theta_{ijg} + \log s_{j}\}\\ &\quad+ \sum\limits_{i=1}^{n} \sum\limits_{i=g}^{G} z_{ig} (\boldsymbol{\theta}_{ig} + \log \mathbf{s}) \mathbf{y}_{i}^{\prime} \\ & \,-\, \sum\limits_{i=1}^{n} \sum\limits_{g=1}^{G} \sum\limits_{j=1}^{d} z_{ig} \log \mathbf{y}_{ij} ! \,-\, \frac{nd}{2} \log 2 \pi \,-\, \frac{1}{2} \sum\limits_{g=1}^{G} \!n_{g} \log | \mathbf{\Sigma}_{g} |\\ &- \frac{1}{2} \sum\limits_{i=1}^{n} \sum\limits_{g=1}^{G} z_{ig} (\boldsymbol{\theta}_{ig} - \boldsymbol{\mu}_{g}) \mathbf{\Sigma}_{g}^{^{\raisebox{.2ex}{\(\scriptscriptstyle-1\)}}} (\boldsymbol{\theta}_{ig} - \boldsymbol{\mu}_{g})^{\prime}, \end{aligned}} $$

where $n_{g} = \sum \nolimits _{i=1}^{n} z_{ig}^{(t)}$. The conditional expectation of complete-data log-likelihood given observed data ($\mathcal {Q}$) is

1 $$  \begin{aligned} \mathcal{Q}(\boldsymbol{\Theta}) & = \mathbb{E} \left[ l_{c}(\boldsymbol{\Theta}) \right] = \mathbb{E} \left[ \log \big(\pi_{g} f(\boldsymbol{y}|\boldsymbol{\theta}_{g}, \boldsymbol{s}) f(\boldsymbol{\theta}_{g} | \boldsymbol{\vartheta}_{g}) \big) \right]. \end{aligned}  $$

Here, ***𝜗***_*g*_=(***μ***_*g*_,***Σ***_*g*_), for *g*=1,…,*G*. Because the first term of (1) does not depend on parameters $\boldsymbol {\vartheta }_{g}, \mathcal {Q}$ can be written

2 $$  \begin{aligned} \mathcal{Q}(\boldsymbol{\vartheta}_{g}| \boldsymbol{\vartheta}_{g}^{(t)}) & = \mathbb{E} \left[\log f(\boldsymbol{\theta}_{g}| \boldsymbol{Y}, \boldsymbol{\vartheta}_{g}) | \boldsymbol{Y} = \boldsymbol{y} \right] + c(\boldsymbol{y}), \end{aligned}  $$

where *c* is independent of ***𝜗***_*g*_. The density of the term *f*(***θ***_*g*_|***y***,***𝜗***_*g*_) in (2) is

3 $$  {\begin{aligned} f(\boldsymbol{\theta}_{g} | \boldsymbol{y}, \boldsymbol{\vartheta}_{g}) & = \frac{f(\boldsymbol{y} | \boldsymbol{\theta}_{g}) f(\boldsymbol{\theta}_{g}, \boldsymbol{\vartheta}_{g})} {f(\boldsymbol{y}, \boldsymbol{\vartheta}_{g})} = \frac{f(\boldsymbol{y} | \boldsymbol{\theta}_{g}) f(\boldsymbol{\theta}_{g}, \boldsymbol{\vartheta}_{g})} {\int_{\boldsymbol{\theta}_{g}} f(\boldsymbol{y} | \boldsymbol{\theta}_{g}) f(\boldsymbol{\theta}_{g}, \boldsymbol{\vartheta}_{g}) \mathrm{d} \boldsymbol{\theta}_{g}}. \end{aligned}}  $$

Due to the integral present in (3), evaluation of *f*(***y***,***𝜗***_*g*_) is difficult. Therefore, the E-step cannot be solved analytically. Here, an extension of the EM algorithm, called Monte Carlo EM (MCEM) [36], can be used to approximate the $\mathcal {Q}$ function. MCEM involves simulating at each iteration *t* and for each observation ***y***_*i*_ a random sample of size *B*, i.e., $\boldsymbol {\theta }^{(1)}_{ig}, \ldots,\boldsymbol {\theta }^{(B)}_{ig}$, from the distribution *f*(***θ***_*g*_|***y***,***𝜗***_*g*_) to find a Monte Carlo approximation to the conditional expectation of complete-data log-likelihood given observed data. Here, each iteration from the MCEM simulation is represented using *k*, where *k*=1,…,*B*. As the values from initial iterations are discarded from further analysis to minimize bias, the number of iterations used for parameter estimation is *N*, where *N*<*B*. Thus, a Monte Carlo approximation for $\mathcal {Q}$ in (2) is

$$\begin{aligned} \mathcal{Q}(\boldsymbol{\vartheta}_{g}| \boldsymbol{\vartheta}_{g}^{(t)}) & =\sum\limits_{g=1}^{G} \sum\limits_{i=1}^{n} \mathcal{Q}_{ig}(\boldsymbol{\vartheta}_{g}| \boldsymbol{\vartheta}_{g}^{(t)}),\\ \mathcal{Q}_{ig}(\boldsymbol{\vartheta}_{g}| \boldsymbol{\vartheta}_{g}^{(t)}) & \simeq \frac{1}{N} \sum\limits_{k=1}^{N} \log f(\boldsymbol{\theta}_{ig}^{(k)}|\boldsymbol{y}_{i},\boldsymbol{\vartheta}_{g}) + c(\boldsymbol{y}_{i}). \end{aligned} $$

However, another layer of complexity is added as the distribution of *f*(***θ***_*g*_|***y***,***𝜗***_*g*_) is unknown. Therefore, an alternative MCEM based on Markov chains, Markov chain Monte Carlo expectation-maximization (MCMC-EM) is proposed. MCMC-EM is implemented via Stan, which is a probabilistic programming language written in C++. The R interface of Stan is available via RStan.

### Bayesian Inference With Stan

Bayesian approaches to mixture modeling offer the flexibility of sampling from computationally complex models using MCMC algorithms. For the mixtures of MPLN distributions, the random sample $\boldsymbol {\theta }^{(1)}_{ig}, \ldots,\boldsymbol {\theta }^{(B)}_{ig}$ is simulated via the RStan package. RStan carries out sampling from the posterior distribution via No-U-Turn Sampler (NUTS). The prior on ***θ***_*ig*_ is a multivariate Gaussian distribution and the likelihood follows a Poisson distribution. Within RStan, the warmup argument is set to half the number of total iterations, as recommended [37]. The warmup samples are used to tune the sampler and are discarded from further analysis.

Using MCMC-EM, the expected value of ***θ***_*ig*_ and group membership variable *Z*_*ig*_, respectively, are updated in E-step as follows

$$\begin{aligned} &\mathbb{E}(\boldsymbol{\theta}_{ig} |\mathbf{y}_{i}) \simeq \frac{1}{N} \sum\limits_{k=1}^{N} \boldsymbol{\theta}_{ig}^{(k)} \simeq \boldsymbol{\theta}^{(t)}_{ig},\\ &\mathbb{E}(Z_{ig} |\mathbf{y}_{i}, \boldsymbol{\theta}_{ig}, \mathbf{s}) = \frac{ \pi_{g} f\left(\mathbf{y}_{i}| \boldsymbol{\theta}_{ig}^{(t)}, \mathbf{s}\right) f\left(\boldsymbol{\theta}_{ig}|\boldsymbol{\mu}_{g}^{(t)}, \boldsymbol{\Sigma}_{g}^{(t)}\right)}{\sum\nolimits_{h=1}^{G} \pi_{h}^{(t)} f(\boldsymbol{y}_{i}|\boldsymbol{\theta}_{ih}^{(t)}, \mathbf{s}) f(\boldsymbol{\theta}_{ih}|\boldsymbol{\mu}_{h}^{(t)}, \boldsymbol{\Sigma}_{h}^{(t)})} =: z_{ig}^{(t)}. \end{aligned} $$

During the M-step, the updates of the parameters are obtained as follows

$$ \begin{aligned} &\pi_{g}^{(t+1)} = \frac{\sum\nolimits_{i=1}^{n} z_{ig}^{(t)}}{n},\qquad \boldsymbol{\mu}^{(t+1)}_{g} = \frac{\sum\nolimits_{i=1}^{n} z_{ig}^{(t)} \mathbb{E} (\boldsymbol{\theta}_{ig})} {\sum\nolimits_{i=1}^{n} z_{ig}^{(t)}},\\ &\boldsymbol{\Sigma}^{(t+1)}_{g} = \frac{\sum\nolimits_{i=1}^{n} z_{ig}^{(t)} \mathbb{E} \left(\left(\boldsymbol{\theta}_{ig} - \boldsymbol{\mu}^{(t+1)}_{g}\right) \left(\boldsymbol{\theta}_{ig} - \boldsymbol{\mu}^{(t+1)}_{g}\right)^{\prime} \right)} {\sum\nolimits_{i=1}^{n} z_{ig}^{(t)} }. \end{aligned} $$

### Convergence

To determine whether the MCMC chains have converged to the posterior distribution, two diagnostic criteria are used. One is the *potential scale reduction factor* [38] and the other is the *effective number of samples* [39]. The algorithm for mixtures of MPLN distributions is set to check if the RStan generated chains have a *potential scale reduction factor* less than 1.1 and an *effective number of samples* value greater than 100 [37]. If both criteria are met, the algorithm proceeds. Otherwise, the chain length is set to increase by 100 iterations and sampling is redone. A total of 3 chains are run at once, as recommended [37]. The Monte Carlo sample size should be increased with the MCMC-EM iteration count due to persistent Monte Carlo error [40], which can contribute to slow or no convergence. For the algorithm for mixtures of MPLN distributions, the number of RStan iterations is set to start with a modest number of 1000 and is increased with each MCMC-EM iteration as the algorithm proceeds. To check if the likelihood has reached its maximum, the Heidelberger and Welch’s convergence diagnostic [41] is applied to all log-likelihood values after each MCMC-EM iteration, using a significance level of 0.05. The diagnostic is implemented via the heidel.diag function in coda package [42]. If not converged, further MCMC-EM iterations are performed until convergence is reached.

### Initialization

For initialization of parameters ***μ***_*g*_ and ***Σ***_*g*_, the mean and cov functions in R are applied to the input dataset, respectively, and log of the resulting values are used. For initialization of $\hat {z}_{ig}$, two algorithms are provided: *k*-means and random. For *k*-means initialization, *k*-means clustering is performed on the dataset and the resulting group memberships are used for the initialization of $\hat {z}_{ig}$. The mixtures of MPLN algorithm is then run for 10 iterations and the resulting $\hat {z}_{ig}$ values are used as starting values. For random initialization, random values are chosen for $\hat {z}_{ig} \in [0,1]$ such that $\sum \nolimits _{i=1}^{n} \hat {z}_{ig} = 1$ for all *i*. The mixtures of MPLN algorithm is then run for 10 iterations and resulting $\hat {z}_{ig}$ values are used as starting values. If multiple initialization runs are considered, the $\hat {z}_{ig}$ values corresponding to the run with the highest log-likelihood value are used for downstream analysis. The value of the fixed, known constant that accounts for the differences in library sizes, **s**, is calculated using the calcNormFactors function from the edgeR package [43].

### Parallel Implementation

Coarse grain parallelization has been developed in the context of model-based clustering of Gaussian mixtures [44]. When a range of clusters are considered for a dataset, i.e., *G*_min_: *G*_max_, each cluster size, *G*, is independent and there is no dependency between them. Therefore, each *G* can be run in parallel, each one on a different processor. Here, the algorithm for mixtures of MPLN distributions is parallelized using parallel package [45] and foreach package [46]. Parallelization reduced the running time of the datasets (results not shown) and all analyses were done using the parallelized code.

### Model selection

The Bayesian information criterion (BIC) [47] remains the most popular criterion for model-based clustering applications [8]. For this analysis, four model selection criteria were used: the Akaike information criterion (AIC) [48],

$$\text{AIC} = -2 \log \mathcal{L} (\hat{\boldsymbol{\vartheta}} |\boldsymbol{y}) + 2K;$$

the BIC,

$$\text{BIC} = -2 \log \mathcal{L} (\hat{\boldsymbol{\vartheta}} |\boldsymbol{y}) + K \log (n);$$

a variation on the AIC used by [49],

$$\text{AIC3} = -2 \log \mathcal{L} (\hat{\boldsymbol{\vartheta}}|\boldsymbol{y}) + 3K;$$

and the integrated completed likelihood (ICL) of [50],

$$\text{ICL} \approx \text{BIC} + 2 \sum\limits_{i=1}^{n} \sum\limits_{g=1}^{G} \text{MAP}\{ \hat{z}_{ig}\} \log \hat{z}_{ig}.$$

The $\mathcal {L} (\hat {\boldsymbol {\vartheta }} |\mathbf {y})$ represents maximized log-likelihood, $\hat {\boldsymbol {\vartheta }}$ is the maximum likelihood estimate of the model parameters ***𝜗***, *n* is the number of observations, and $\text {MAP}\{ \hat {z}_{ig}\}$ is the maximum *a posteriori* classification given $\hat {z}_{ig}$. *K* represents the number of free parameters in the model, calculated as *K*=(*G*−1)+(*Gd*)+*Gd*(*d*+1)/2, for *G* clusters. These model selection criteria differ in terms of how they penalize the log-likelihood. Rau et al. [14] make use of an alternative approach to model selection using slope heuristics [51, 52]. Following their work, Djump and DDSE, available via capushe package, were also used. More than 10 models need to be considered for applying slope heuristics.

## Additional files

Additional file 1 Expression patterns of different models. The expression patterns for different models of cranberry RNA-seq dataset. (PDF 1631 kb)

Additional file 2 GO analysis of different models. GO enrichment analysis results for the different models selected for cranberry RNA-seq dataset. (XLSX 17 kb)

Additional file 3 Parameter estimation results of simulated data. Parameter estimation results of mu and sigma values for simulated data using mixtures of MPLN distributions. (PDF 77 kb)

## Data Availability

The RNA-seq dataset used for transcriptome data analysis is available on the NCBI SRA under the BioProject PRJNA380220 https://www.ncbi.nlm.nih.gov/bioproject/PRJNA380220/. All scripts used for implementing the mixtures of MPLN algorithm and simulation data can be found at https://github.com/anjalisilva/MPLNClust.

## Abbreviations

AIC: Akaike information criterion
AIC3: Bozdogan Akaike information criterion
ARI: Adjusted Rand index
BIC: Bayesian information criterion
D: Darkening
DDSE: Data-driven slope estimation
Djump: Dimension jump
E: Early
EM: Expectation-maximization
GO: Gene ontology
I: Intermediate
ICL: Integrated completed likelihood
M: Mature
MAP: Maximum a posteriori probability
MCEM: Monte Carlo expectation-maximization
MCMC-EM: Markov chain Monte Carlo expectation-maximization
MPLN: Multivariate Poisson-log normal
NB: Negative binomial
NCBI: National Center for Biotechnology Information
ND: Non-darkening
NUTS: No-U-Turn Sampler
RNA-seq: RNA sequencing
SRA: Sequence Read Archive
TMM: Trimmed mean of *M* values
