# Supplementary material for: A multivariate Poisson-log normal mixture model for clustering transcriptome sequencing data
Source: BMC Bioinformatics. 2019 Jul 16;20:394. doi: 10.1186/s12859-019-2916-0 (PMC6636065; doi:10.1186/s12859-019-2916-0)
Supplement: Supplementary file 1 — Expression patterns of different models. The expression patterns for different models of cranberry RNA-seq dataset. (PDF 1631 kb) [file 12859_2019_2916_MOESM1_ESM.pdf]

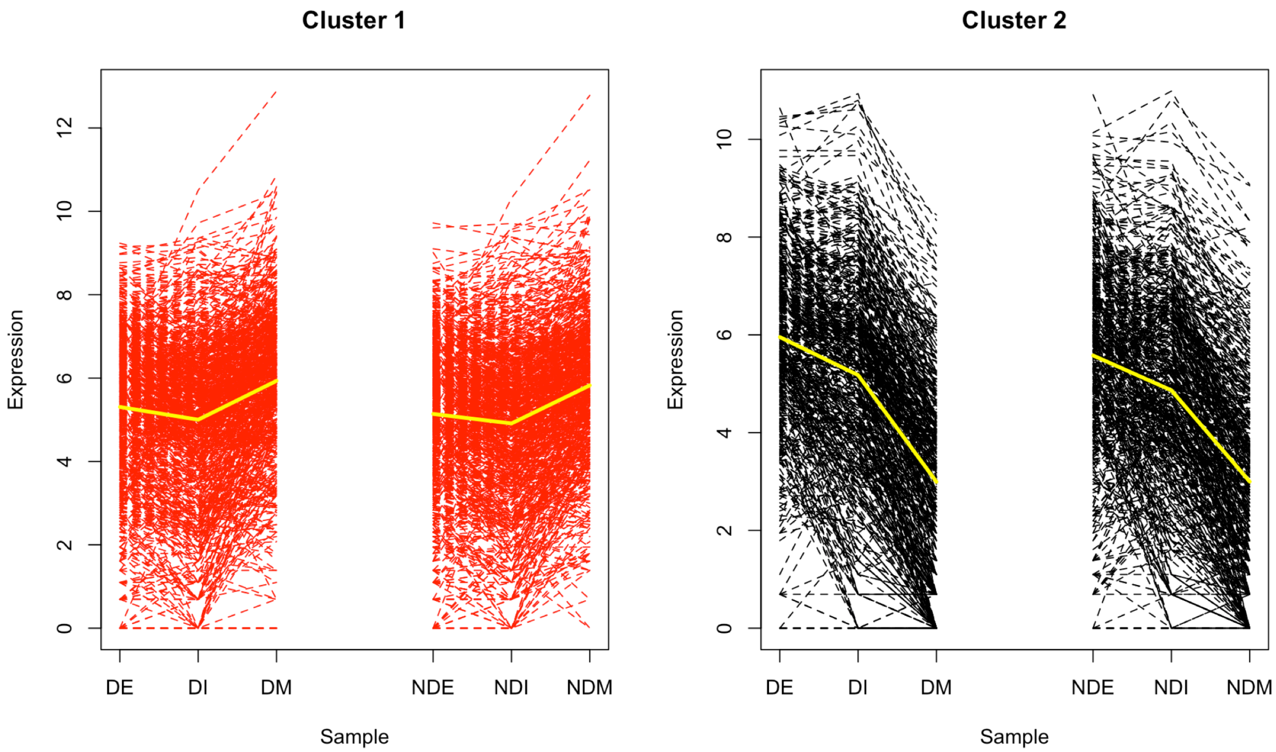

Figure 1: The expression patterns for the $G = 2$ model for the cranberry bean RNA-seq dataset clustered using mixtures of `MBCluster.Seq,NB.` in T_1.

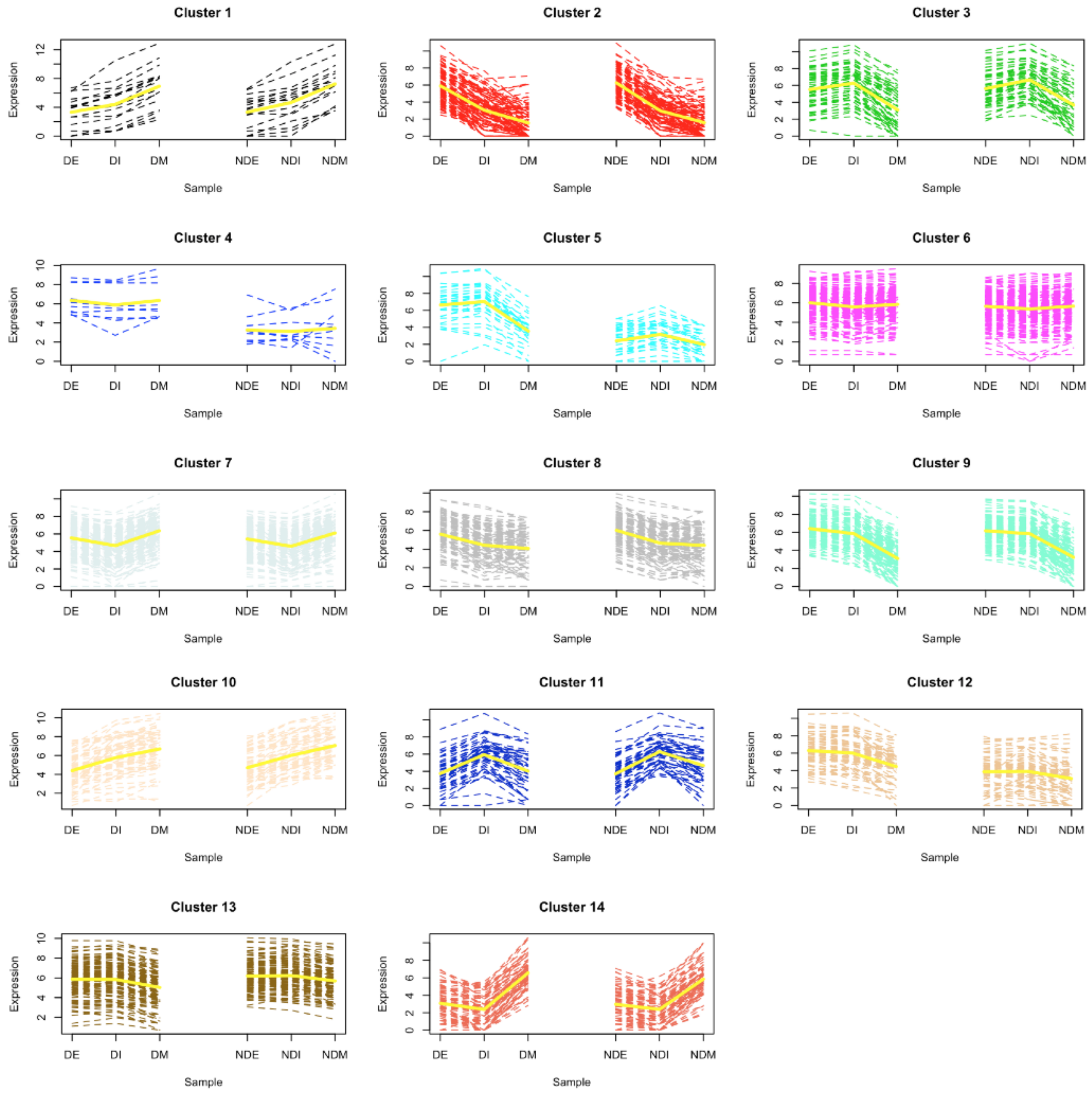

Figure 2: The expression patterns for the $G = 14$ model for the cranberry bean RNA-seq dataset clustered using mixtures of `MBCluster.Seq,Poisson` in T_2.
