# Supplementary material for: A multivariate Poisson-log normal mixture model for clustering transcriptome sequencing data
Source: BMC Bioinformatics. 2019 Jul 16;20:394. doi: 10.1186/s12859-019-2916-0 (PMC6636065; doi:10.1186/s12859-019-2916-0)
Supplement: Supplementary file 3 — Parameter estimation results of simulated data. Parameter estimation results of mu and sigma values for simulated data using mixtures of MPLN distributions. (PDF 77 kb) [file 12859_2019_2916_MOESM3_ESM.pdf]

1 Parameter Estimation Results

Table 1: Model parameters ($\boldsymbol{\vartheta}_g$) and the norms of the associated biases from the 50 runs for $G = 1$.

$\boldsymbol{\vartheta}_g$	True						$\|\|$Bias$\|\|$
$\boldsymbol{\mu}_1$	$(4.5, 5, 5, 5, 5, 6)'$						0.06
	0.97	−0.28	−0.40	−0.04	−0.42	−0.30	
	−0.28	1.00	0.08	0.18	0.68	−0.59	
	−0.40	0.08	0.97	−0.35	−0.01	0.03	
$\boldsymbol{\Sigma}_1$	−0.04	0.18	−0.35	0.90	0.66	0.30	0.04
	−0.42	0.68 − 0.01	0.66	1.06	0.16		
	−0.30	−0.59	0.03	0.30	0.16	1.02	

Table 2: Model parameters ($\boldsymbol{\vartheta}_g$) and the norms of the associated biases from the 50 runs for $G = 2$.

$\boldsymbol{\vartheta}_g$	True						$\|\|$Bias$\|\|$
$\boldsymbol{\mu}_1$	$(6.5, 6, 6, 6, 6, 6)'$						0.11
$\boldsymbol{\mu}_2$	$(2, 2.5, 2, 2, 2, 2)'$						0.13
	1.24	−0.36	−0.51	−0.04	−0.54	−0.39	
	−0.36	1.30	0.11	0.23	0.90	−0.77	
	−0.51	0.11	1.25	−0.44	−0.01	0.04	
$\boldsymbol{\Sigma}_1$	−0.04	0.23	−0.44	1.09	0.84	0.38	0.05
	−0.54	0.90	−0.01	0.84	1.41	0.21	
	−0.39	−0.77	0.04	0.38	0.21	1.33	
	0.70	0.26	−0.45	−0.30	−0.04	−0.14	
	0.26	0.70	0.19	0.27	−0.07	−0.05	
	−0.45	0.19	0.70	0.29	0.09	0.13	
$\boldsymbol{\Sigma}_2$	−0.30	0.27	0.29	0.70	0.25	−0.04	0.15
	−0.04	−0.07	0.09	0.25	0.70	0.02	
	−0.14	−0.05	0.13	−0.04	0.02	0.70	

Table 3: Model parameters ($\boldsymbol{\vartheta}_g$) and the norms of the associated biases from the 30 runs for $G = 3$.

$\boldsymbol{\vartheta}_g$	True	$\lVert\text{Bias}\rVert$
$\boldsymbol{\mu}_1$	$(3, 3, 3, 3, 3, 3)'$	0.15
$\boldsymbol{\mu}_2$	$(6.5, 6.5, 6.5, 6.5, 6, 6.5)'$	0.13
$\boldsymbol{\mu}_3$	$(1, -1, 1, 1, -1, 1)'$	0.73
$\boldsymbol{\Sigma}_1$	$\begin{bmatrix} 1.00 & -0.29 & -0.41 & -0.04 & -0.41 & -0.31 \\ -0.29 & 1.00 & 0.08 & 0.19 & 0.66 & -0.59 \\ -0.41 & 0.08 & 1.00 & -0.38 & -0.01 & 0.03 \\ -0.04 & 0.19 & -0.38 & 1.00 & 0.67 & 0.31 \\ -0.41 & 0.66 & -0.01 & 0.67 & 1.00 & 0.15 \\ -0.31 & -0.59 & 0.03 & 0.31 & 0.15 & 1.00 \end{bmatrix}$	1.55
$\boldsymbol{\Sigma}_2$	$\begin{bmatrix} 1.50 & -0.03 & 0.67 & 0.66 & -0.65 & -1.06 \\ -0.03 & 1.50 & -0.01 & 0.52 & 0.14 & -0.58 \\ 0.67 & -0.01 & 1.50 & 0.64 & 0.28 & -0.44 \\ 0.66 & 0.52 & 0.64 & 1.50 & 0.56 & -0.96 \\ -0.65 & 0.14 & 0.28 & 0.56 & 1.50 & 0.41 \\ -1.06 & -0.58 & -0.44 & -0.96 & 0.41 & 1.50 \end{bmatrix}$	0.12
$\boldsymbol{\Sigma}_3$	$\begin{bmatrix} 0.50 & 0.30 & -0.09 & -0.06 & 0.04 & -0.04 \\ 0.30 & 0.50 & -0.02 & -0.02 & -0.07 & -0.17 \\ -0.09 & -0.02 & 0.50 & 0.09 & 0.26 & 0.13 \\ -0.06 & -0.02 & 0.09 & 0.50 & -0.01 & 0.19 \\ 0.04 & -0.07 & 0.26 & -0.01 & 0.50 & -0.10 \\ -0.04 & -0.17 & 0.13 & 0.19 & -0.10 & 0.50 \end{bmatrix}$	0.20
